# Cost-Effectiveness of Domestic PD-1 Inhibitor Camrelizumab Combined With Chemotherapy in the First-Line Treatment of Advanced Nonsquamous Non–Small-Cell Lung Cancer in China

**DOI:** 10.3389/fphar.2021.728440

**Published:** 2021-11-02

**Authors:** Liu Qiao, Zhen Zhou, Xiaohui Zeng, Chongqing Tan

**Affiliations:** ^1^ Department of Pharmacy, The Second Xiangya Hospital of Central South University, Changsha, China; ^2^ Menzies Institute for Medical Research, University of Tasmania, Hobart, TAS, Australia; ^3^ PET-CT Center, The Second Xiangya Hospital of Central South University, Changsha, China

**Keywords:** cost-effectiveness, nonsquamous non–small-cell lung cancer, camrelizumab, pembrolizumab, pemetrexed, China

## Abstract

**Objective:** Camrelizumab is the first domestic PD-1inhibitor approved to be combined with chemotherapy as a first-line therapy for advanced nonsquamous non–small-cell lung cancer (NSCLC) in China. The purpose of this study was to determine whether using camrelizumab in the first-line setting is cost-effective in China when compared with traditional chemotherapy or the imported PD-1inhibitor pembrolizumab.

**Material and Methods:** A Markov model was built to simulate 3-week patient transitions over a 30-year horizon from the perspective of the Chinese healthcare system. Health states included stable disease, first progression, second progression, and death. A direct comparison between first-line camrelizumab in combination with pemetrexed and carboplatin (CPC) and pemetrexed plus carboplatin (PC) was performed by calculating transition probabilities from the CameL trial. An indirect comparison between first-line CPC and pembrolizumab in combination with pemetrexed and platinum (PPP) was performed by calculating transition probabilities using a network meta-analysis. Costs in the Chinese setting were collected from the local public database and literatures. Sensitivity analyses explored the uncertainty around model parameters.

**Results:** In the primary analysis, first-line CPC gained an additional 0.41 quality-adjusted life-years (QALYs) with an incremental cost of $3,486 compared with PC, resulting in an incremental cost-effectiveness ratio (ICER) of $8,378 per QALY gained. In the secondary analysis, first-line PPP yielded an additional 0.10 QALYs at an incremental cost of $6,710, resulting in an ICER of $65,563 per QALY gained.

**Conclusion:** For Chinese patients with advanced nonsquamous NSCLC without targetable genetic aberrations, our primary analysis results supported first-line CPC as a cost-effective treatment compared with traditional PC chemotherapy. The findings of our secondary analysis suggested that first-line PPP would not be a cost-effective option compared with first-line CPC. This analysis provided strong evidence for promoting the widespread use of first-line CPC in China and, to some extent, stimulated the enthusiasm for the development of domestic cancer drugs.

## Introduction

In China, first-line platinum-doublet chemotherapy remained the category one recommendation for advanced nonsquamous non–small-cell lung cancer (NSCLC) without targetable genetic aberrations (Zhou et al., 2021a), although immunotherapy with remarkable efficacy has been approved by the Chinese National Medical Products Administration (NMPA) in recent years (Gandhi et al., 2018; Wu et al., 2021). Pembrolizumab (a programmed death receptor 1 [PD-1] inhibitor) monotherapy was the first immunotherapy approved as the mainstay for treating advanced nonsquamous NSCLC without targetable genetic aberrations in October 2019 in China, followed by pembrolizumab in combination with pemetrexed and platinum (PPP), approved in December 2019 (National Medical Products Administration and CenterFor Drug Evaluation, 2019). Although pembrolizumab monotherapy and its combination therapy have brought considerable survival benefits to Chinese patients with advanced nonsquamous NSCLC, the imported PD-1inhibitor pembrolizumab costs more than $84,000 per year, which limits its widespread use in China with a per capita gross domestic product (GDP) of about $10,000 (National Bureau Of Statistics Of China, 2020). In 2013, Chinese national-level medical spending was obviously ahead of those of all BRICS and G7 members except the United States (US) (Jakovljevic, 2016) and is expected to grow steadily over the next decade, making the allocation of limited resources a core challenge in China. In term of cancer treatments, the cost-effectiveness of a cutting-edge treatment option is the most key determinant to justify its widespread use.

In June 2020, camrelizumab, a domestic PD-1 inhibitor, was launched in China as a new first-line therapeutic option for advanced nonsquamous NSCLC without targetable genetic aberrations (National Medical Products Administration, 2020). The approval of camrelizumab in combination with pemetrexed and carboplatin (CPC) was in response to the result of a phase three clinical trial (CameL) to evaluate its efficacy against nonsquamous NSCLC without targetable genetic aberrations in China (Zhou et al., 2021b). This trial demonstrated that compared with pemetrexed plus carboplatin (PC), first-line CPC significantly prolonged the progression-free survival (PFS) by a median of 3 months (median, 11.3 vs. 8.3 months). The median overall survival (OS) in the CPC-treated group was estimated to be 27.9 months, the longest OS that has been recorded in clinical trials of first-line immunotherapies for advanced nonsquamous NSCLC without targetable genetic aberrations in the world so far (Zhou et al., 2021b). Given its favorable net benefits, camrelizumab has successfully occupied a place in the National Reimbursement Drug List (NRDL) in China, with the annual cost decreasing from $53,000 to $ 8,000 (Human resources and Social Security Department of National Medical Insurance Bureau, 2020).

As the first domestic PD-1 inhibitor approved as a first-line therapy for treating advanced nonsquamous NSCLC without targetable genetic aberration, camrelizumab offers a great opportunity for reducing the healthcare expenditures on cancer at both national and individual levels. In 2015, approximately 60% of 623,000 newly diagnosed NSCLC cases presented with metastatic diseases, of which nearly three-quarters (approximately 280,000 cases) were classified as having the nonsquamous histologic type (Chen et al., 2016). Due to the huge cancer burden and limited medical resources in China, the enthusiasm surrounding new therapies with superior efficacy must be balanced against their potential financial consequences (Shi et al., 2016). Thus, cost-effectiveness analyses are needed to evaluate whether a new therapy can provide favorable clinical effects at an acceptable cost so as to determine its wider application. As is reflected in the Guidelines of the Chinese Society of Clinical Oncology (CSCO) for NSCLC in 2020, the level of recommendation of chemotherapy alone as the first-line treatment for advanced nonsquamous NSCLC without targetable genetic aberration is higher than that of CPC (Zhou et al., 2021a). The lack of authoritative cost-effectiveness evidence related to camrelizumab may be the key reason why it is not recommended preferentially. Therefore, the primary objective of our study was to evaluate the cost-effectiveness of CPC compared with PC chemotherapy alone in first-line treatment of advanced nonsquamous NSCLC patients without targetable genetic aberration from the perspective of the Chinese healthcare system. In an additional exploratory analysis, we evaluated the cost-effectiveness of the first domestic PD-1 inhibitor camrelizumab versus the first imported PD-1 inhibitor pembrolizumab in the first-line treatment of advanced nonsquamous NSCLC patients without targetable genetic aberration from the perspective of the Chinese healthcare system.

## Materials and Methods

This economic evaluation used a Markov model to estimate the cost-effectiveness of first-line CPC for treating advanced nonsquamous NSCLC patients without targetable genetic aberration from the perspective of the Chinese healthcare system. This economic evaluation was deemed exempt from ethical review as only existing and nonidentifiable data were used, which include clinical efficacy and safety data from the CameL trial and cost data from published literature and local public databases. The study followed the Guidelines for pharmacoeconomic evaluation in China (Chinese Pharmaceutical Association, 2020).

### Patients and Treatment

The primary analysis evaluated two first-line treatment strategies, CPC and PC. Model patients mirrored participants who were enrolled in the CameL trial (ClinicalTrials.gov number, NCT03134872). In the secondary analysis, an indirect first-line treatment comparison between CPC and PPP was performed. Model patients in the CPC group and the PPP group mirrored participants who were enrolled in the CameL trial and the KEYNOTE-189 trial (ClinicalTrials.gov number, NCT02578680), respectively.

First-line dosage and administration schedules followed those detailed in the CameL and KEYNOTE-189 trials. To simplify the model, platinum treatment in the PPP group was modeled as carboplatin because clinicians prefer to use carboplatin rather than cisplatin for treating lung cancer, given its lower toxicity. In addition, folic acid and vitamin B12 were administrated to reduce the toxicity caused by pemetrexed treatment (Zhou et al., 2021b).

After the first progression, based on the Guidelines for NSCLC in China, individuals in the CPC or PPP arm could subsequently receive second-line docetaxel chemotherapy, while individuals in the PC arm could subsequently receive second-line nivolumab (Wu et al., 2019; Zhou et al., 2021a). After the second progression, subsequent third-line therapy, including immunotherapy, targeted therapy, and chemotherapy, was provided to patients as long as there were continuous benefits. Supplementary Table S1 provides detailed information on the first-line and subsequent second-line treatments.

### Markov Model

**FIGURE 1:**
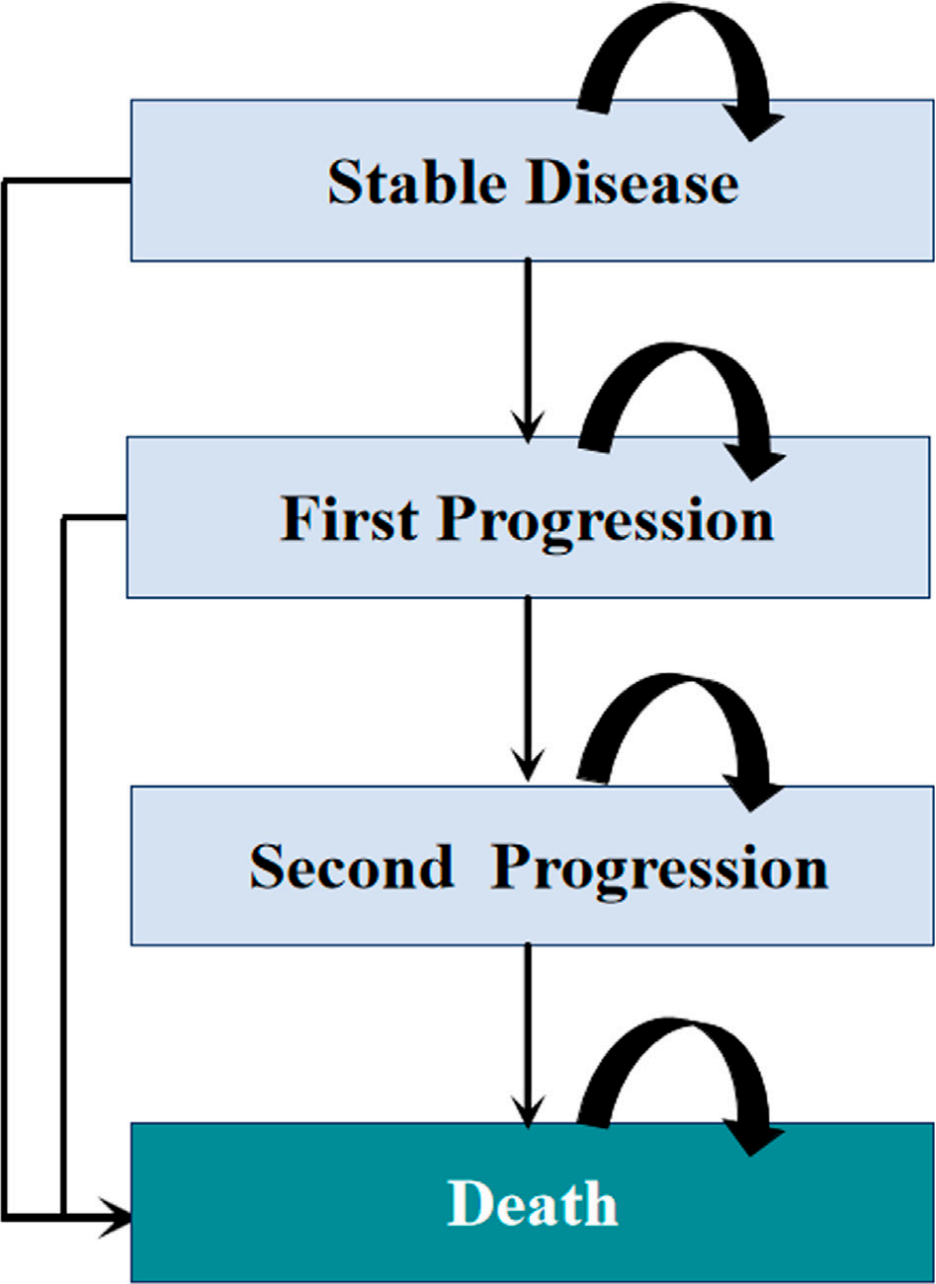
Diagram of the Markov model. The percentage of patients receiving each line of subsequent therapy was defined according to the CameL and KEYNOTE-189 clinical trials.

Model patients were simulated through four health states: stable disease, first progression, second progression, and death (Figure 1). Patients were initially in the stable disease state and could receive first-line treatments until first progression, death, or other causes. Patients who experienced disease progression could then receive subsequent therapy, as long as there were sustained benefits; otherwise, they were provided the best support care (BSC). The proportion of patients receiving each line of subsequent therapy was adopted from the CameL and KEYNOTE-189 trials (Supplementary Table S2). In line with the Guidelines for NSCLC in China, patients were recommended for end-of-life care before death (Zhou et al., 2021a). Supplement Figure S1 summarizes the treatment strategies used in this analysis**.**

In our Markov model, the cycle length was set to 3 weeks in alignment with the treatment schedule. The model was used to estimate costs and effectiveness [measured by quality-adjusted life-years (QALYs)] for each treatment strategy over a 30-year time horizon. The cost-effectiveness of a strategy relative to an alternative strategy was assessed by comparing the incremental cost-effectiveness ratios (ICERs) reflecting the incremental cost for each QALY gained, with the willingness-to-pay (WTP) threshold. In this analysis, the WTP threshold was defined as 1× China’s per capita GDP in 2020 (Ochalek et al., 2020), that is, $11,146 per QALY (National Bureau Of Statistics Of China, 2020). Both costs and effectiveness were discounted at an annual rate of 5%. This economic evaluation was conducted using TreeAge Pro software (version 2021, https://www.treeage.com/) to build the Markov model and R software (version 4.0.4, http://www.r-project.org) to perform parametric survival modeling.

### Transition Probabilities and Health State Utilities

In the primary analysis, the Kaplan–Meier (KM) survival curves from the CameL trial were used to estimate transition probabilities from the stable disease state to death and from the stable disease state to the first progression state for the first-line CPC and PC. First, we extracted OS and PFS data points from the corresponding KM survival curves using the GetData Graph Digitizer software package (version 2.26; http://www.getdata-graphdigitizer.com/index.php). Second, pseudo-individual patient data were generated using the algorithm proposed by Hoyle and Henley (2011) to minimize the difference between the target data (the CameL trial) and the modeled data (Hoyle and Henley, 2011). Third, log-logistic distribution provided the best fit to the recreated survival data and was therefore used to predict survival beyond the CameL trial period (Supplementary Figure S2). Finally, the log-logistic distribution parameters, theta (θ) and kappa (κ), were computed using R software (Table1). The survival probability at time *t* was calculated as follows: $\text{S}\left(\text{t}\right)=1/\left[1+\text{exp}\left(\text{θ}\right){\text{t}}^{\text{κ}}\right]$ . The transition probability from the stable disease state to the first progression state at a given time *t* was calculated as follows: $\text{P}\left(\text{t}\right)=1-\left[1+\text{exp}\left(\text{θ}\right){\left(\text{t}-1\right)}^{\text{κ}}\right]/\left[1+\text{exp}\left(\text{θ}\right){\text{t}}^{\text{κ}}\right]$ . In the secondary analysis, the HRs of OS and PFS for PPP versus CPC were estimated by a network meta-analysis implemented using the Gemtc package of R software. The survival probability at time *t* for first-line PPP was calculated as follows: $\text{S}\left(\text{t}\right)={\left[1/\left[1+\text{exp}\left(\text{θ}\right){\text{t}}^{\text{κ}}\right]\right]}^{{\text{HR}}_{\text{ OS}}}$ . By analogy, the transition probability from the stable disease state to the first progression state at a given time *t* was calculated as follows: $\text{P}\left(\text{t}\right)=1-{\left[1+\text{exp}\left(\text{θ}\right){\left(\text{t}-1\right)}^{\text{κ}}\right]}^{{\text{HR}}_{\text{ PFS}}}/{\left[1+\text{exp}\left(\text{θ}\right){\text{t}}^{\text{κ}}\right]}^{{\text{HR}}_{\text{ PFS}}}$ . The estimation of transition probabilities from the first progression state to death and from the first progression state to the second progression state was based on the PFS and OS KM curves related to second-line nivolumab and docetaxel reported in the CheckMate 078 trial (Wu et al., 2019), using the same method as described above and reported in our previous study (Liu et al., 2020a). A background mortality rate for each age group was identified from Chinese life tables (Supplementary Table S3) (World Health Organization, 2020).

**TABLE 1:** Model inputs.

Variable	Baseline value	Range	Distribution	Source
Survival				
Log-logistic OS survival model with CPC	Theta = 0.003326; kappa = 2.439,689	—	—	[7]
Log-logistic OS survival model with PC	Theta = 0.004501; kappa = 1.618,052	—	—	[7]
Log-logistic PFS survival model with CPC	Theta = 0.01503; kappa = 1.46627	—	—	[7]
Log-logistic PFS survival model with PC	Theta = 0.03905; kappa = 1.36768	—	—	[7]
OS HR network meta-analysis of PPP versus CPC	0.71	0.57–0.85	Normal	Estimated^a^
PFS HR network meta-analysis of PPP versus CPC	0.92	0.74–1.10	Normal	Estimated^a^
Weibull OS survival model with second-line docetaxel	Scale = 0.07471; shape = 1.15519	—	—	[12]
Weibull PFS survival model with second-line docetaxel	Scale = 0.25070; shape = 1.14230	—	—	[12]
OS HR of second-line nivolumab versus docetaxel	0.76	0.56–1.04	Normal	[12]
PFS HR of second-line nivolumab versus docetaxel	0.87	0.46–1.19	Normal	[12]
Costs (US$)				
Camrelizumab (200 mg per cycle)	424.51	212.25–636.76	Gamma	Local charge
Pembrolizumab (200 mg per cycle)	2,597.79	1,298.89–3,896.69	Gamma	Local charge
Pemetrexed (500 mg/m^2^ per cycle)	79.31	39.65–118.96	Gamma	Estimated^b^
Nivolumab (4.5 mg/kg per cycle)	60.35	30.17–90.52	Gamma	Local charge
Docetaxel (75 mg/m^2^ per cycle)	5.61	2.81–8.41	Gamma	Estimated^b^
Routine follow-up cost per cycle	55.60	27.80–83.40	Gamma	[20]
Subsequent third-line therapy cost per cycle	854.05	427.02–1,281.08	Gamma	[20]
BSC cost per cycle	337.50	168.75–506.25	Gamma	[20]
End-of-life care cost per cycle	2,627.80	1,313.90–3,941.70	Gamma	[20]
AEs cost for CPC	1,407.53	703.77–2,111.30	Gamma	Estimated^c^
AEs cost for PPP	1,141.48	570.74–1712.22	Gamma	Estimated^c^
AEs cost for PC	883.90	441.95–1,325.85	Gamma	Estimated^c^
Utilities				
Stable disease	0.856	0.718–0.994	Beta	[17]
First progression	0.768	0.595–0.941	Beta	[17]
Second progression	0.703	0.545–0.861	Beta	[17]
Disutility for CPC	0.079	0.061–0.096	Beta	Estimated^c^
Disutility for PPP	0.045	0.035–0.056	Beta	Estimated^c^
Disutility for PC	0.063	0.049–0.077	Beta	Estimated^c^
Other				
Discount rate (%)	5	0–8	Fixed in PSA	[1]
Patient weight (kg)	65	52–78	Normal	[20]
Body surface area (m2)	1.72	1.38–2.07	Normal	[20]

OS, overall survival; PFS, progression-free survival; HR, hazard ratio; CPC, camrelizumab in combination with pemetrexed and carboplatin; PC, pemetrexed plus carboplatin; PPP, pembrolizumab in combination with platinum; BSC, best supportive care; AEs, adverse events.

a Based on our network meta-analysis.

b Pemetrexed and docetaxel have been included in the National Reimbursement Drug List, and 80% of the drug expenses incurred by pemetrexed and 95% of the drug expenses incurred by docetaxel in the treatment of advanced nonsquamous non–small-cell lung cancer can be reimbursed through medical insurance.

c Estimated in Supplemental Table S4.

For all model groups, the health state utilities were based on a utility value assessment for Chinese patients with NSCLC using the EuroQol five-dimension (EQ-5D) instrument and the Chinese-specific value algorithm (Shen et al., 2018). We incorporated in the model the utility decreases caused by treatment-related grade III/IV toxicities; the decline in utility was derived from the literature (Nafees et al., 2017). Detailed information on health state utilities is provided in Table 1 and Supplementary Table S4.

### Costs

We considered the costs of first-line and subsequent treatment, treating adverse events (AEs), and general treatment associated with disease management including routine follow-up, BSC, and end-of-life care. In the first-line and subsequent second-line treatments, the price of camrelizumab, pembrolizumab, and nivolumab were sourced from the big data service platform for China’s health industry (https://www.yaozh.com/) (The big data service platform for China’s health industry, 2021). According to the cancer immunotherapy patient assistance program in China, NSCLC patients could avail up to 2 years of assistance after purchasing four cycles of pembrolizumab. In terms of this, four cycle’s cost of pembrolizumab was considered in our model. In calculating dosage amounts, we used a mean body weight of 65 kg and a mean body surface area of 1.72 m^2^ for base case patients (Liu et al., 2020b). In the context of the universal medical insurance systems, essential drugs such as carboplatin, folic acid, and vitamin B12 have been fully covered by the National Reimbursement Drug List (NRDL), and the proportion of patient’s out-of-pocket expenses for these drugs is 0%. Therefore, the costs of these drugs were excluded from this analysis. Besides, pemetrexed and docetaxel have been included in the NRDL, with a reimbursement proportion of 80 and 95%, respectively.

In addition, to better reflect the cost of first-line and second-line treatments in real-world settings, the duration of these treatments were adjusted based on the median treatment cycles reported in the respective clinical trials (Gandhi et al., 2018; Wu et al., 2019; Zhou et al., 2021b), to account for the fact that patients may discontinue first-line and second-line treatments due to unacceptable toxicity, consent withdrawal, or investigator decision, in addition to progression and death. The cost of subsequent third-line therapy, routine follow-up, BSC, and end-of-life care came from a published study (Liu et al., 2020b).

The cost of commonly reported grade III/IV AEs with an incidence of >5% were incorporated in the model, including neutropenia, thrombocytopenia, and anemia (Gandhi et al., 2018; Zhou et al., 2021b). Although some common immune-related AEs related to camrelizumab were reported, such as reactive capillary endothelial proliferation and immune-related pneumonitis, their costs were not considered in this model because of their low grade III/IV incidence. The costs per patient corresponding to each AE were sourced from published literature (Supplement Table S4) (Gu et al., 2019; You et al., 2019). Cost inputs are detailed in Table 1.

### Sensitivity Analysis

To assess the uncertainty around model parameters, deterministic sensitivity analysis (DSA) and probabilistic sensitivity analysis (PSA) were conducted. During DSA, model parameters varied individually within the ranges detailed in Table 1 to ascertain their impact on the ICERs. Utility values and HRs were tested within their respective 95% CIs, costs were tested within ±50% of baseline values, and other variables were tested within plausible ranges available from published literatures. During PSA, model parameters varied simultaneously to verify the robustness of our findings. Estimates of 1,000 ICERs were generated by running Monte Carlo simulations with random sampling from the distribution of each model parameter. Utility values were described by beta distributions, costs by gamma distributions, and HRs, patient weight, and body surface area by normal distributions.

## Results

### Incremental Cost-Effectiveness Ratios

In the primary analysis, first-line CPC prolonged survival by 0.41 QALYs (1.57 vs. 1.16 QALYs), which was approximately equivalent to 5 months of perfect health, while increasing health care costs by $3,486 ($11,519 vs. $8,082) compared to the first-line PC. Therefore, the ICER between CPC and PC was estimated to be $8,378 per QALY gained (Table 2).

**TABLE 2:** Summary of simulation results.

Analysis	Cost, $	QALYs	Incremental		ICER, $/QALY
			Cost, $	QALYs	
Primary					
PC	8,082	1.16			
CPC	11,519	1.57	3,438	0.41	8,378
Secondary					
CPC	11,519	1.57			
PPP	18,230	1.67	6,710	0.10	65,563

QALY, quality-adjusted life-years; ICER, incremental cost-effectiveness ratios; CPC, camrelizumab in combination with pemetrexed and carboplatin; PC, pemetrexed plus carboplatin; PPP, pembrolizumab in combination with platinum.

In the secondary analysis, first-line PPP was associated with a mean cost of $18,230 and a mean survival of 1.67 QALYs. Compared with first-line CPC, first-line PPP yielded an additional 0.10 QALYs at an incremental cost of $ 6,710, resulting in an ICER of 65,563 per QALY gained, which was almost 5 times higher than the WTP threshold ($11,146/QALY) set for this analysis (Table 2).

### Sensitivity Analysis

**FIGURE 2:**
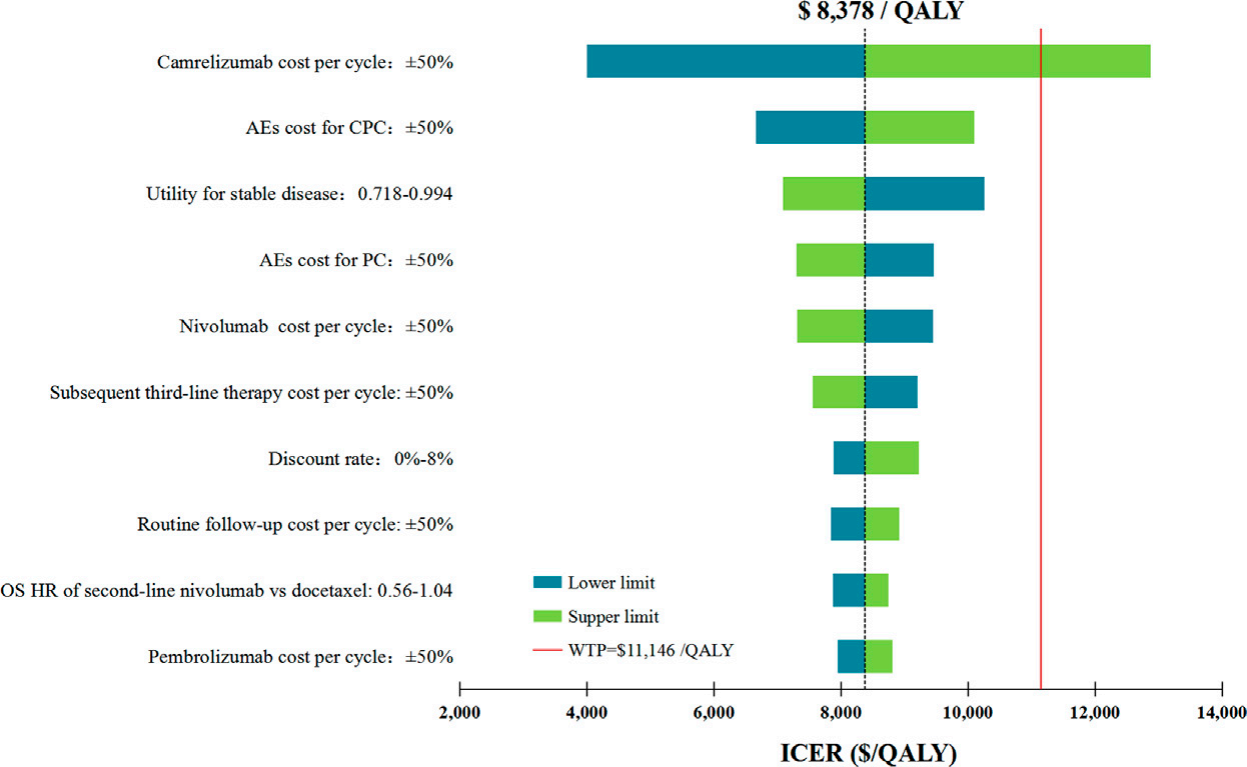
Deterministic sensitivity analysis for the primary analysis. The red solid line represents the willingness-to-pay threshold of $11,146/QALY used in this study. The black dotted line represents the ICER of $ 8,378/QALY in the primary analysis. The top 10 variables by magnitude of effect are shown. ICER indicated incremental cost-effectiveness ratios; QALY, quality-adjusted life-years; AEs, adverse events; OS, overall survival; CPC, camrelizumab in combination with pemetrexed and carboplatin; PC, pemetrexed plus carboplatin.

In performing DAS for the primary analysis, any of the tested model parameters were unable to change the cost-effective treatment strategy from CPC to PC, except the camrelizumab cost per cycle. For instance, increasing the camrelizumab cost per cycle from $424.51 to more than $553.92 resulted in the ICERs between CPC and PC being above the WTP threshold. The upper limit of AE cost for CPC and the lower limit of utility for stable disease make the ICERs close to the WTP threshold, which were $10,094/QALY and $10,262/QALY, respectively. Other model parameters had little effect on the ICER for CPC vs. PC (Figure 2).

In performing DAS for the secondary analysis, first-line PPP was cost-effective only at the low limits of the PFS HR of first-line PPP vs. CPC. Large decreases in the ICER also occurred at the low limit of the pembrolizumab cost per cycle ($12,897/QALY). Other model parameters varied but did not substantially change the results (Supplementary Figure S3. The PAS of the primary analysis revealed that the probability of first-line CPC being cost-effective was 36% at the WTP threshold of $11,146/QALY, and this probability increased with the rising WTP thresholds (Supplementary Figure S4). In the secondary analysis, first-line PPP was not cost-effective in any of the 1,000 iterations at the WTP threshold of $11,146/QALY.

## Discussion

Using a Markov model, we studied the cost-effectiveness of camrelizumab, the first domestic PD-1 inhibitor in the first-line treatment of advanced nonsquamous NSCLC patients without targetable genetic aberrations. In our primary analysis, we found that first-line CPC is a cost-effective option in comparison with first-line PC, with an ICER of $8,378/QALY below the WTP threshold of $11,146/QALY. In our secondary analysis, we compared the cost-effectiveness for the first domestic PD-1 inhibitor camrelizumab versus the first imported PD-1 inhibitor pembrolizumab and found that the first-line PPP was unlikely to be a cost-effective treatment strategy compared with first-line CPC due to the unfavorable ICER exceeding the WTP threshold by 5 times.

Sensitivity analyses conducted for the primary analysis suggested that the camrelizumab cost per cycle was the only model parameter that substantially changed our result. To the best of our knowledge, after the official establishment of the National Healthcare Security Administration (NHSA) in May 2018, several rounds of negotiations with pharmaceutical companies on the price of cancer drugs were immediately launched, with the purpose of solving the medical burden of cancer patients through national strategic procurement (National Healthcare Security Administration, 2018). Camrelizumab was successfully negotiated and officially incorporated into the NRDL in March 2021, and its price dropped from $14.35/mg to $2.12/mg (Human resources and Social Security Department of National Medical Insurance Bureau, 2020). In this context, a rising trend in the camrelizumab price is unlikely; our findings were therefore sufficient to support the use of first-line CPC as a cost-effective regimen for advanced nonsquamous NSCLC patients without targetable genetic aberrations.

The key finding of our secondary analysis is that the first-line PPP may be associated with increased health care costs by improving PFS that required more expensive treatment. This conclusion was supported by our sensitivity analyses showing that the model was most affected by PFS HR of first-line PPP vs. CPC, and a HR value lower than 0.81 would allow the first-line PPP to be cost-effective. In the current cost-effectiveness analysis, HRs for PPP vs. CPC estimated by our network meta-analysis was used to perform an indirect comparison due to the lack of clinical trials with head-to-head comparisons. Adjusting the HRs instead of directly using the clinical data from different trials is expected to provide more reliable results. Furthermore, the minor difference in QALYs suggested that long-term efficacy between the two first-line regimes may be similar among target patients. Our conclusions regarding the poor cost-effectiveness of first-line PPP may add important evidence to promote the widespread use of first-line CPC.

To our knowledge, this is the first cost-effectiveness study of domestic versus imported PD-1 inhibitors in the first-line setting of patients with advanced nonsquamous NSCLC in China. As the prices of domestic cancer drugs are considerably lower than those of imported cancer drugs, cost-effectiveness analyses on them have important implications for reducing national health expenditure. The growing trend of the cancer epidemic has imposed a heavy economic burden on the healthcare system in China (Global Burden of Disease Cancer Collaboration et al., 2018; Jakovljevic et al., 2019a). Despite the increasing number of Chinese people who can afford the imported treatment, it is still challenging for the majority of cancer patients to pay out of pocket, leading them to be poorly treated or untreated (Jakovljevic, 2015). Therefore, delivering low-cost cutting-edge treatment options to cancer patients may serve as a feasible strategy for the Chinese government to be more effective in the management of the healthcare system (Jakovljevic et al., 2019b). Our current findings have certain significance for incentivizing the Chinese government to expand their investment into the research and development of novel domestic cancer drugs.

This study has several notable strengths. First, we maximized the use of the latest clinical trial’s data through economic modeling to estimate costs and outcomes associated with the three first-line treatment strategies over a 30-year horizon. Second, we incorporated the real-world performance in the second-line treatment of advanced nonsquamous NSCLC patients without targetable genetic aberrations, such as the use of nivolumab for patients with front-line chemotherapy failure and the use of docetaxel in individuals for whom front-line immunotherapy had failed (Zhou et al., 2021a). Third, the median treatment cycles with regard to first-line and second-line drugs were considered in our model to illustrate that in clinical practice, in addition to progression and death, patients may discontinue treatment due to unacceptable toxicity, consent withdrawal, and investigator decision, and so on (Zhou et al., 2021b). Fourth, our model considered three lines of treatments, as well as the BSC and end-of-life care in our model, in order to provide a complete picture of the treatment pattern in this patient population which may be closer to the real clinical practice than clinical trials.

This study also has several limitations. First, the cost-effectiveness between first-line PPP and CPC was indirectly compared by synthesizing efficacy and safety data from two clinical trials (the CameL and KEYNOTE-189 trials). Although the adjusted HRs used to estimate transition probabilities were obtained by employing a network meta-analysis, there is uncertainty regarding whether different races lead to significantly different responses to therapies. Second, health state utilities used in this study were derived from published literature due to the fact that the quality-of-life data in the CameL trial were unavailable. We tested the robustness of our model and found that varying the health state utilities in sensitivity analysis did not substantially change our results. Third, most cost inputs that populated our model were derived from local sources except for the general treatment costs associated with disease management and subsequent third-line therapy, which were informed by published studies. However, our results seemed insensitive to these costs. Fourth, some novel therapies also approved as a standard first-line treatment, such as bevacizumab in combination with paclitaxel and carboplatin, were not considered in the current analysis. The main obstacles lied in the potential heterogeneity between the Chinese patient populations recruited in the CameL trial and the BEYOND trial, which evaluated the efficacy of first-line BCP (Zhou et al., 2015). Differing from the BEYOND trial with no requirement on targetable genetic aberrations, the CameL trial specifically focused on NSCLC patients without EGFR and ALK alteration. Therefore, future studies are expected to confirm the cost-effectiveness for CPC versus BPC when the clinical data are mature.

In conclusion, for Chinese patients with advanced nonsquamous NSCLC without targetable genetic aberrations, results of our primary analysis supported first-line CPC as a cost-effective treatment compared with traditional PC chemotherapy. The findings of our secondary analysis suggested that the first-line PPP would not be a cost-effective option compared with first-line CPC. This analysis provided strong evidence for promoting the widespread use of first-line CPC in China and, to some extent, stimulated the enthusiasm for the development of domestic cancer drugs.

## Data Availability

The original contributions presented in the study are included in the article/Supplementary Material; further inquiries can be directed to the corresponding authors.
