# Supplementary material for: Cost-Effectiveness of Domestic PD-1 Inhibitor Camrelizumab Combined With Chemotherapy in the First-Line Treatment of Advanced Nonsquamous Non–Small-Cell Lung Cancer in China
Source: Front Pharmacol. 2021 Nov 2;12:728440. doi: 10.3389/fphar.2021.728440 (PMC8593416; doi:10.3389/fphar.2021.728440)
Supplement: Supplementary file 1 [file Table1.docx]

Table 1. First-line and subsequent second-line treatments in the model.

| **Regimens** | **Dose Sizez** | **Treatment schedule** | **Median exposure duration**  **(3-week cycles)** |
| --- | --- | --- | --- |
| First-line  Camrelizumab+  Pemetrexed+  Carboplatin | camrelizumab, 200mg | every 3-week cycles up to 35 cycles | 10 cycles |
|  | pemetrexed, 500mg/m^2^ | four to six 3-week cycles | 10 cycles |
|  | carboplatin, AUC 5.0mg/ml/min | four to six 3-week cycles | 5 cycles |
| First-line  Pembrolizumab+  Pemetrexed+  Carboplatin | pembrolizumab,200mg | every 3-week cycles up to 35 cycles | 10.5 cycles |
|  | pemetrexed, 500mg/m^2^ | four 3-week cycles | 9.5 cycles |
|  | carboplatin, AUC 5.0mg/ml/min | four 3-week cycles | 3.6 cycles |
| First-line  Pemetrexed+  Carboplatin | pemetrexed, 500mg/m^2^ | four to six 3-week cycles | 7 cycles |
|  | carboplatin, AUC 5.0mg/ml/min | four to six 3-week cycles | 4 cycles |
| Second-line  Nivolumab | nivolumab, 3 mg/kg | every 2 weeks | 4 cycles |
| Second-line  docetaxel | Docetaxel,75mg/m^2^ | every 3 weeks | 2 cycles |

*AUC, Area under curve.*
