# Supplementary figures and images for: Cost-Effectiveness of Domestic PD-1 Inhibitor Camrelizumab Combined With Chemotherapy in the First-Line Treatment of Advanced Nonsquamous Non–Small-Cell Lung Cancer in China
Source: Front Pharmacol. 2021 Nov 2;12:728440. doi: 10.3389/fphar.2021.728440 (PMC8593416; doi:10.3389/fphar.2021.728440)

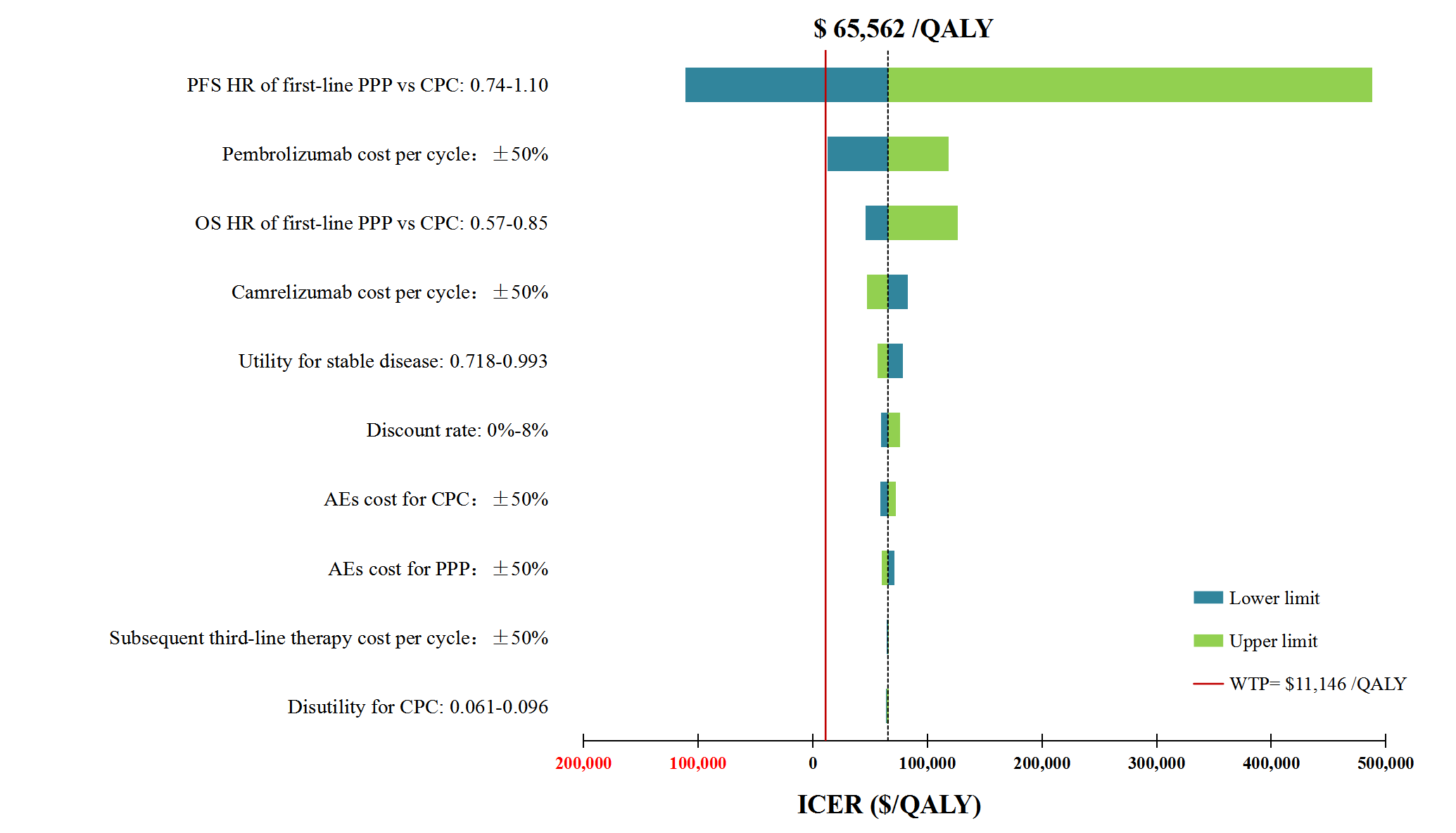

Supplement: Supplementary file 2 [file Image3.tif]

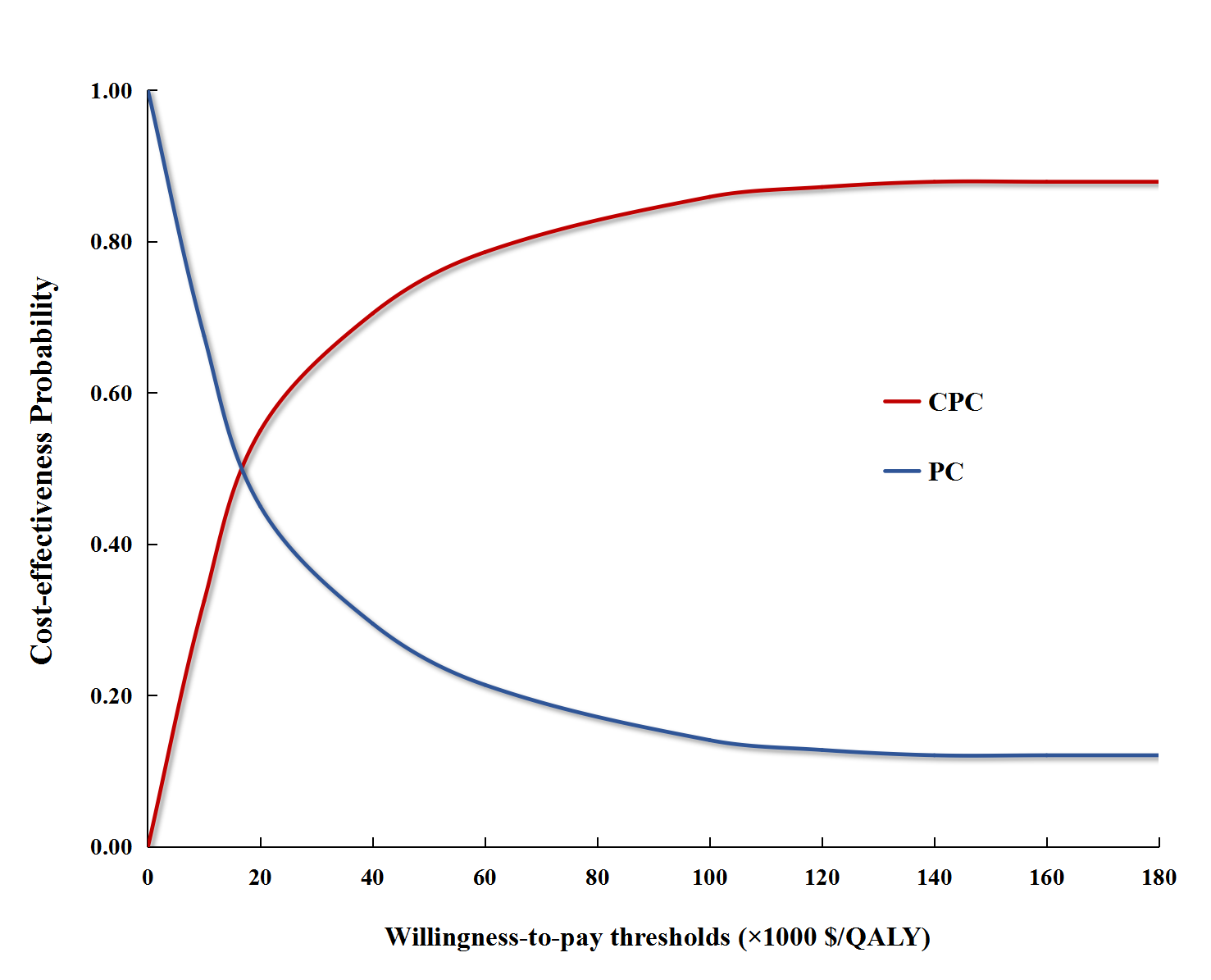

Supplement: Supplementary file 3 [file Image4.tif]

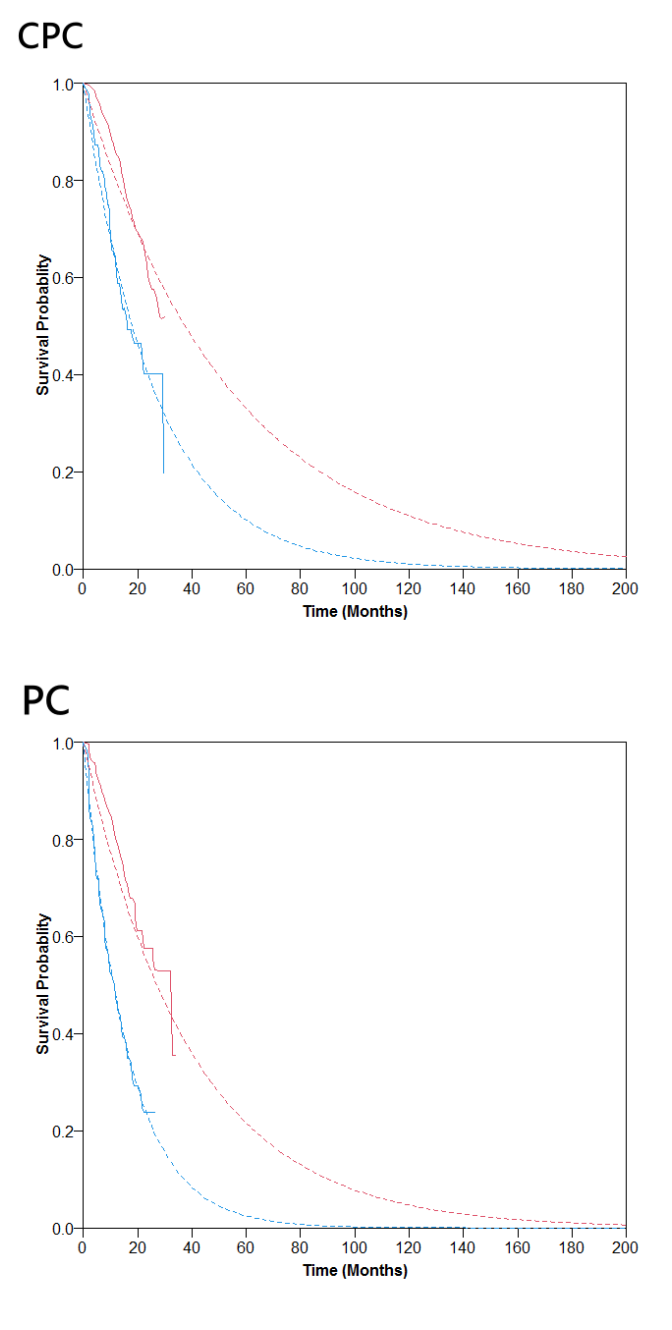

Supplement: Supplementary file 5 [file Image2.tif]

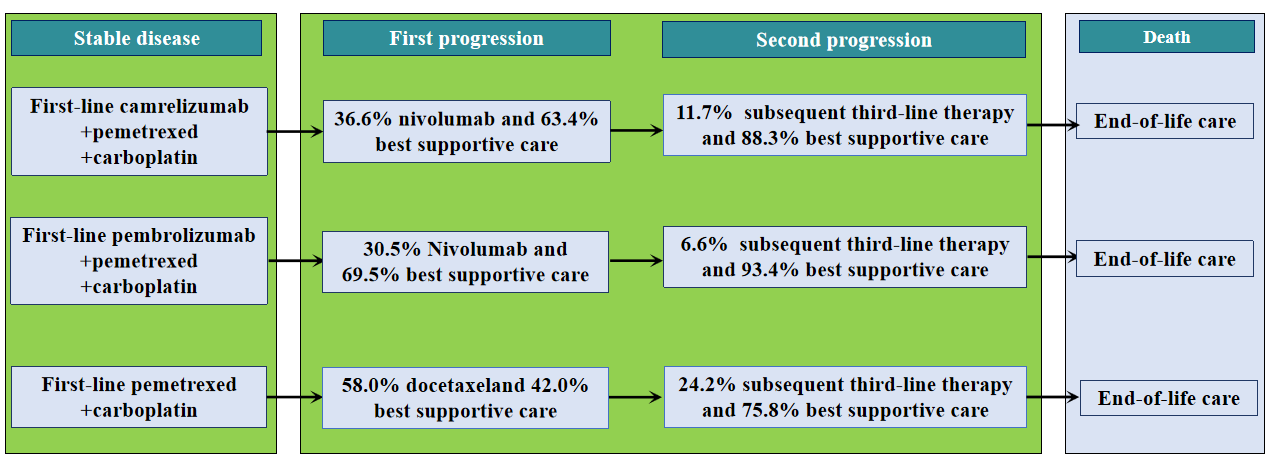

Supplement: Supplementary file 6 [file Image1.tif]
