# Supplementary material for: Cost-Effectiveness of Domestic PD-1 Inhibitor Camrelizumab Combined With Chemotherapy in the First-Line Treatment of Advanced Nonsquamous Non–Small-Cell Lung Cancer in China
Source: Front Pharmacol. 2021 Nov 2;12:728440. doi: 10.3389/fphar.2021.728440 (PMC8593416; doi:10.3389/fphar.2021.728440)
Supplement: Supplementary file 4 [file DataSheet1.ZIP › Source data/HRs for PPP vs CPC (R language and results).docx]

OS:

library("gemtc")

library("rjags")

treatments <-read.table(textConnection('

id description

1 "camrelizumab"

2 "pembrolizumab"

3 "placebo"'), header=TRUE)

data <- read.table(textConnection('

study treatment diff std.err

1 1 -0.3147107 0.1670104

1 3 NA NA

2 2 -0.6539265 0.1528363

2 3 NA NA

'), header=TRUE)

View(data)

network <- mtc.network(data.re=data,description="Example", treatments=treatments)

plot(network)

model<-mtc.model(network,type="consistency", factor = 2.5,n.chain=4,likelihood="binom",link="cloglog",linearModel="random")

results <- mtc.run(model, n.adapt =10000, n.iter = 100000, thin = 10,sampler ="JAGS")

forest(relative.effect(results,"1"))

rank.probability(results,preferredDirection=-1)


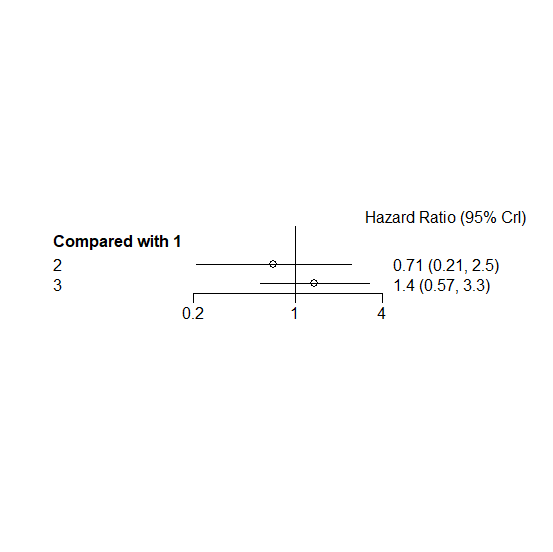


Rank probability; preferred direction = -1

[,1] [,2] [,3]

1 0.226100 0.600175 0.173725

2 0.759475 0.194825 0.045700

3 0.014425 0.205000 0.780575

PFS

library("gemtc")

library("rjags")

treatments <-read.table(textConnection('

id description

1 "camrelizumab"

2 "pembrolizumab"

3 "placebo"'), header=TRUE)

data <- read.table(textConnection('

study treatment diff std.err

1 1 -0.5108256 0.1435677

1 3 NA NA

2 2 -0.597837 0.1184453

2 3 NA NA

'), header=TRUE)

View(data)

network <- mtc.network(data.re=data,description="Example", treatments=treatments)

plot(network)

model<-mtc.model(network,type="consistency", factor = 2.5,n.chain=4,likelihood="binom",link="cloglog",linearModel="random")

results <- mtc.run(model, n.adapt =10000, n.iter = 100000, thin = 10,sampler ="JAGS")

forest(relative.effect(results,"1"))

rank.probability(results,preferredDirection=-1)


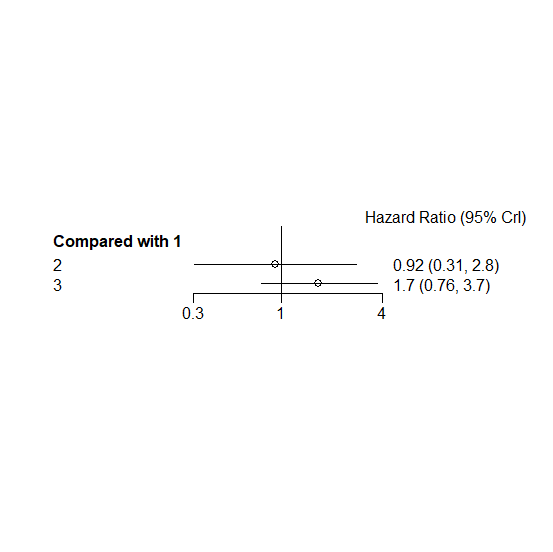


Rank probability; preferred direction = -1

[,1] [,2] [,3]

1 0.410075 0.519750 0.070175

2 0.583400 0.369775 0.046825

3 0.006525 0.110475 0.883000
