# Supplementary material for: Cost-Effectiveness of Domestic PD-1 Inhibitor Camrelizumab Combined With Chemotherapy in the First-Line Treatment of Advanced Nonsquamous Non–Small-Cell Lung Cancer in China
Source: Front Pharmacol. 2021 Nov 2;12:728440. doi: 10.3389/fphar.2021.728440 (PMC8593416; doi:10.3389/fphar.2021.728440)
Supplement: Supplementary file 7 [file Table2.docx]

Table 2. The proportions of patients receiving each line of subsequent therapy.

| **Regimens** | **Subsequent second-line therapy** | **Subsequent third-line therapy** |
| --- | --- | --- |
| Camrelizumab+  Pemetrexed+  Carboplatin | 36.6% | 11.7% |
| Pembrolizumab+  Pemetrexed+  Carboplatin | 30.5% | 6.6% |
| Pemetrexed+  Carboplatin | 58.0% | 24.2% |
