# Supplementary material for: Cost-Effectiveness of Domestic PD-1 Inhibitor Camrelizumab Combined With Chemotherapy in the First-Line Treatment of Advanced Nonsquamous Non–Small-Cell Lung Cancer in China
Source: Front Pharmacol. 2021 Nov 2;12:728440. doi: 10.3389/fphar.2021.728440 (PMC8593416; doi:10.3389/fphar.2021.728440)
Supplement: Supplementary file 8 [file Table3.docx]

Table 3. Background mortality rate for each age group.

| **Age Group** | **Background mortality rate** | |
| --- | --- | --- |
|  | **Male** | **Female** |
| 15-19 years | 0.0024 | 0.0011 |
| 20-24 years | 0.0040 | 0.0016 |
| 25-29 years | 0.0044 | 0.0016 |
| 30-34 years | 0.0061 | 0.0023 |
| 35-39 years | 0.0088 | 0.0034 |
| 40-44 years | 0.0131 | 0.0054 |
| 45-49 years | 0.0170 | 0.0075 |
| 50-54 years | 0.0263 | 0.0127 |
| 55-59 years | 0.0403 | 0.0203 |
| 60-64 years | 0.0634 | 0.0339 |
| 65-69 years | 0.0992 | 0.0572 |
| 70-74 years | 0.1719 | 0.1044 |
| 75-79 years | 0.2721 | 0.1754 |
| 80-84 years | 0.445 | 0.311 |
| 85+ years | 1 | 1 |
