# Supplementary material for: Cost-Effectiveness of Domestic PD-1 Inhibitor Camrelizumab Combined With Chemotherapy in the First-Line Treatment of Advanced Nonsquamous Non–Small-Cell Lung Cancer in China
Source: Front Pharmacol. 2021 Nov 2;12:728440. doi: 10.3389/fphar.2021.728440 (PMC8593416; doi:10.3389/fphar.2021.728440)
Supplement: Supplementary file 9 [file Table4.docx]

Table 4. Proportion, costs and disutility of grade III/IV AEs considered in the model.

| AEs | Proportion (%) | | | Cost per event ($) | Disutility |
| --- | --- | --- | --- | --- | --- |
|  | CPC | PPP | PC |  |  |
| Neutropenia | 38.0% | 15.8% | 30.4% | 466.00 | 0.200 |
| Anaemia | 18.5% | 16.3% | 11.1% | 6434.00 | / |
| Thrombocytopenia | 16.6% | 7.9% | 11.6% | 241.90 | / |
| Diarrhea | / | 5.2% | / | 5.48 | 0.070 |
| Fatigue | / | 5.7% | / | Not considered in the model^a^ | 0.070 |
| Vomiting | 1.0% | 3.7% | 1.9% | Not considered in the model^a^ | 0.120 |
| Rash | 1.5% | 1.70% | 0.0% | Not considered in the model^a^ | 0.100 |
| Estimated AEs Costs and disutility | | | |  |  |
| AEs cost for CPC, $ | | | | 1407.53 |  |
| AEs cost for PPP, $ | | | | 1141.48 |  |
| AEs cost for PC, $ | | | | 883.90 |  |
| AEs disutility for CPC | | | |  | 0.079 |
| AEs disutility for PPP | | | |  | 0.045 |
| AEs disutility for PC | | | |  | 0.063 |

*AEs, adverse events; CPC, camrelizumab in combination with pemetrexed and carboplatin; PPP, pembrolizumab in combination with platinum;PC, pemetrexed plus carboplatin;.*

*^a^The cost of commonly reported grade III/IV AEs with an incidence of >5% were incorporated the model. However, fatigue may be an exception because it is unlikely to cost treatment, according to the clinical practice.*
